# Control of multidrug resistant bacteria in a tertiary care hospital in India

**DOI:** 10.1186/2047-2994-1-23

**Published:** 2012-06-06

**Authors:** Namita Jaggi, Pushpa Sissodia, Lalit Sharma

**Affiliations:** 1 Labs & Infection Control, Artemis Health Institute, Sector 51, Gurgaon, Haryana-122001, India; 2 Laboratory Services, Artemis Health Institute, Sector 51, Gurgaon, Haryana-122001, India

**Keywords:** Carbapenem resistance, Gram negatives, Antimicrobial stewardship program, DDD and Antimicrobial resistance

## Abstract

**Background:**

The objective of this study was to assess the impact of antimicrobial stewardship programs on the multidrug resistance patterns of bacterial isolates. The study comprised an initial retrospective analysis of multidrug resistance in bacterial isolates for one year (July 2007-June 2008) followed by prospective evaluation of the impact of Antimicrobial Stewardship programs on resistance for two years and nine months (July 2008-March 2011).

**Setting:**

A 300-bed tertiary care private hospital in Gurgaon, Haryana (India)

**Findings:**

**Methods:**

Study Design

• July 2007 to June 2008: Resistance patterns of bacterial isolates were studied.

• July 2008: Phase I intervention programme Implementation of an antibiotic policy in the hospital.

• July 2008 to June 2010: Assessment of the impact of the Phase I intervention programme.

• July 2010 to March 2011: Phase II intervention programme: Formation and effective functioning of the antimicrobial stewardship committee. Statistical correlation of the Defined daily dose (DDD) for prescribed drugs with the antimicrobial resistance of Gram negatives.

**Results:**

Phase I intervention programme (July 2008) resulted in a decrease of 4.47% in ESBLs (*E.coli* and *Klebsiella*) and a significant decrease of 40.8% in carbapenem-resistant *Pseudomonas*. Phase II intervention (July 2010) brought a significant reduction (24.7%) in carbapenem-resistant *Pseudomonas.* However, the resistance in the other Gram negatives *(E.coli*, *Klebsiella*, and *Acinetobacter)* rose and then stabilized. A positive correlation was observed in *Pseudomonas* and *Acinetobacter* with carbapenems and cefoperazone-sulbactam.

Piperacillin-tazobactam showed a positive correlation with *Acinetobacter* only. *E.coli* and *Klebsiella* showed positive correlation with cefoparazone-sulbactam and piperacillin-tazobactam.

**Conclusion:**

An antimicrobial stewardship programme with sustained and multifaceted efforts is essential to promote the judicious use of antibiotics.

## Findings

### Introduction

Antimicrobial resistance is not a new phenomenon; however, the current magnitude and the speed with which it is developing is a cause for global concern including in India.

Multidrug resistant organisms (MDROs) are defined as microorganisms that are resistant to one or more classes of antimicrobial agents (e.g., ESBL, MRSA, VRE etc.) [1]. These highly resistant organisms deserve special attention in healthcare facilities as they are associated with increased lengths of stay, costs, and mortality [1]. They can also be transmitted between patients and healthcare workers and lead to the spread of antimicrobial resistance. In most instances, MDRO infections have clinical manifestations that are similar to infections caused by susceptible pathogens. However, options for treating these infections are often extremely limited.

The current need is to develop a robust antimicrobial stewardship programme which would enhance clinical outcomes, reduce non-judicious use of antibiotics and healthcare costs and minimize adverse effects of antimicrobial use (toxicity and resistance). Furthermore, an effective infection control program should be put in place for reducing the transmission of drug-resistant bacteria within the hospital. This should be developed at the local level in hospitals and a national level. A start has been made in India in terms of developing a National Policy for Containment of Antimicrobial Resistance by the Directorate General of Health Services in which a special task force has been created to fulfill the objectives above.

## Materials and methods

The present study was conducted in a 300-bed tertiary care private hospital in Gurgaon, Haryana (India). The bacterial culture data of all samples was analyzed for a total period of 45 months (3 years and 9 months) (July 2007 to March 2011) in the Microbiology laboratory of the hospital. Standard culture methods were used (Practical Medical Microbiology 14th ed. by Colle and Fraser) and the isolates, both Gram positive and Gram negative were processed for identification and antibiotic susceptibility tests using VITEK® 2 Compact system (bioMérieux, Marcy l’Etoile, France), following CLSI guidelines [2-4]. The antibiogram of each confirmed isolate was studied and susceptibility results were compiled using the WHONET 5.4 programme.

Our study was divided into four stages:

July 2007 to June 2008: Resistance patterns of Gram-negative isolates- *E.coli*, *Klebsiella*, *Pseudomonas* and *Acinetobacter* were studied.

July 2008: Phase I intervention programme: Implementation of an antibiotic policy in the hospital. In addition, Infection control practices, such as hand hygiene, a clean environment, appropriate infection barriers and early identification and isolation of patients infected with a transmissible microorganism were also promoted through regular training sessions of the healthcare staff.

July 2008 to June 2010: Assessment of the impact of the Phase I intervention programme.

July 2010 to March 2011: Phase II intervention programme: This included the formation and effective functioning of the antimicrobial stewardship committee.

Defined daily dose (DDD) per 1,000 inpatient-days for each drug (Cefoparazone/Sulbactam, Piperacillin/Tazobactam and Carbapenems{imipenem and meropenem}) prescribed every month was calculated according to the World Health Organization (WHO) anatomical therapeutic chemical (ATC) classification system 2009 [5]. This was statistically correlated with the antimicrobial resistance of *E.coli*, *Klebsiella*, *Pseudomonas* and *Acinetobacter* expressed as incidence density/1000 inpatients to determine the significance of the analysis.

## Results and discussion

Infectious diseases continue to be a leading cause of mortality the world over and more so in developing countries with low access to health services (World Health Report 2007) [6].

The results of the bacterial cultures performed over a period of 45 months (July 2007 to March 2011) in the Microbiology lab of a tertiary care private hospital in Gurgaon, Haryana, India are tabulated (Table 1).

**Table 1:** Total Cultures from July 2007 - Mar 2011

**Period**	**Total Samples**	**Break-up**		**Total Positives**	**Gram Negatives**	**Gram Positives**
**July 2007 -March 2011**	28971	Urine	10970 (37.8%)	2035	1785	250
		Blood	9386	920	676	244
		Respiratory	3865	1300	1136	164
		Pus	1601	854	505	349
		Stool	1368	357	354	3
		Fluids	1781	149	83	66
			**28971**	**5615**	4539 (80.8%)	1076 (19.1%)

A total of 28 971 samples were cultured. The break-up into the various sample types showed that urine cultures were the predominant sample (10 970 out of 28 971) representing 37.86% of the total number.

A total of 5615 isolates were obtained from 28 971 cultures, giving a 19.38% culture yield. Out of the total isolates, 4539 were Gram-negative showing a clear preponderance of Gram-negative pathogens in the hospital environment (80.8%). 19.9% comprised the Gram-positive load (1076 out of 5615)**.**

Among the Gram-negative isolates, *E.coli* (43.9%), *Klebsiella* (19.7%), *Pseudomonas* (15.1%) and *Acinetobacter* (9.69%) were the predominant isolates overall. The antibiograms showed the combined extended spectrum β-lactamases (ESBL) prevalence of 55.3% which included both *E.coli* and *Klebsiella* with 54.2% and 57.9% respectively. The prevalence of carbapenem resistance in *Pseudomonas* and *Acinetobacter* was found to be 27.7% and 85% respectively (Table 2).

**Table 2:** Antimicrobial Resistance of Gram-negative Organisms

**Period**	***E.coli***		***Klebsiella***		***E.coli + Kleb***	***Acinetobacter***		***Pseudomonas***	
	**Total Isolates**	**ESBL (%)**	**Total Isolates**	**ESBL (%)**	**ESBL (%)**	**Total Isolates**	**Carbapenem Resistant (%)**	**Total Isolates**	**Carbapenem Resistant (%)**
July 2007- June 2008	278	142	61	40	182 (53.6%)	16	2 (12.5%)	89	19 (21.3%)
		(51%)		(65.7%)					
July 2008- Dec 2008	253	154	108	40	185 (51.2%)	27	17 (63%)	79	10 (12.6%)
		(57.3%)		(37.0%)					
Jan 2009- Dec 2009	601	280	371	168	448 (46.0%)	136	121 (88.9%)	294	97 (32.9%)
		(46.5%)		(45.2%)					
Jan 2010- Jun 2010	320	184	122	93	277 (62.6%)	102	89 (87.2%)	86	30 (34.8%)
		(57.5%)		(76.2%)					
July 2010- Dec 2010	378	225	125	96	321 (63.8%)	116	104 (89.6%)	99	24 (24.2%)
		(59.5%)		(76.8%)					
Jan 2011 - Mar 2011	167	106	111	83	189 (67.9%)	43	41 (95.3%)	42	11 (26.2%)
		(63.4%)		(74.7%)					
Total	1997	1091	898	520	1602 (55.3%)	440	374 (85%)	689	191(27.7%)
		(54.6%)		(57.9%)					

In India, various studies have shown the prevalence of ESBL in *E.coli* and *Klebsiella* to range from 20% to 60% [7-9]. The carbapenem resistance range reported in *Pseudomonas* was 26-43% [10-12] and in *Acinetobacter,* 21-39% [13-15].

Among the Gram positives, *Staphylococcus aureus* (36.8%), Coagulase negative *Staphylococci* (19.8%) and *Enterococci* (38.1%) were the predominant isolates (Additional file 1: Table S1). Of these isolates, the prevalence of Methicillin-resistant *Staphylococcus aureus* (MRSA) was 36.27%, Methicillin-resistant Coagulase-negative *Staphylococci* (MRSE) was 67.75% and Vancomycin-resistant *Enterococci* (VRE) was 6.58% overall (Table 3).

**Table 3:** Antimicrobial Resistance of Gram-positive Organisms

**Period**	***Staphylococcus aureus***		**Coagulase Negative**		**Enterococci**	
			***Staphylococci***			
	**Total**	(MRSA)* (%)	**Total**	(MRSE)**(%)	**Total**	(VRE)*** (%)
	**Isolates**		**Isolates**		**Isolates**	
July 2007-June 2008	53	15 (28.3%)	22	16 (72.72%)	102	4 (3.9%)
July 2008- Dec 2008	48	14 (29.2%)	21	4 (19.04%)	39	6 (15.3%)
Jan 2009- Dec 2009	135	51 (37.7%)	57	49 (85.9%)	107	6 (5.60%)
Jan 2010- Jun 2010	55	19 (34.5%)	49	33 (67.3%)	52	7 (13.4%)
July 2010- Dec 2010	74	26 (35.1%)	41	28 (68.2%)	89	4 (4.49%)
Jan 2011 - Mar 2011	32	19 (59.3%)	24	14 (62.5%)	21	3 (14.28%)
Total	397	144 (36.2%)	214	144 (67.2%)	410	30 (7.3%)

* - Methicillin-resistant *Staphylococcus aureus.*

** - Methicillin-resistant coagulase-negative *Staphylococci.*

*** - Vancomycin-resistant *Enterococci.*

According to our staged intervention plan, the resistance data of Gram-negative isolates (*E.coli*, *Klebsiella*, *Pseudomonas* and *Acinetobacter*) and Gram-positive isolates (*Staphylococcus aureus*, Coagulase-negative *Staphylococci* and *Enterococci*,) were retrospectively analyzed over a one-year period (July 2007 to June 2008). During this period, the overall ESBL prevalence in *E.coli* and *Klebsiella* was 53.6% and the percentage resistance to carbapenems in *Pseudomonas* and *Acinetobacter* was found to be 21.3% and 12.5%, respectively (Table 2). Among the Gram-positive isolates, MRSA showed a prevalence of 28.3%, MRSE 72.72% and VRE 3.9%. This data is shown in Table 3.

## Phase I intervention programme

The Phase I intervention programme under which the antibiotic policy was introduced and implemented was initiated in July 2008. After assessing the impact of the program, it was observed that the resistance patterns of the isolates for the first 6 months (July 2008- Dec 2008) showed a minor decrease of 4.47% in combined ESBL prevalence in *E.coli* and *Klebsiella* and also a significant decrease of 40.8% in carbapenem resistance towards *Pseudomonas*. However, there was a significant increase in resistance in *Acinetobacter*. This could be because of a lowering of infection control standards or the entry of multidrug resistant *Acinetobacter* from other hospitals.

The trend of decreasing resistance continued the following year (2009) for ESBLs (*E.coli* and *Klebsiella*) showing a significant decrease of 14.1% but the next six months (Jan 2010-June 2010) showed an increase of 36% bringing the ESBL prevalence up to 62.6%. The carbapenem resistance in *Pseudomonas* and *Acinetobacter* showed a substantial increase in the year 2009 followed by a minor decrease of 5.7% and 1.9% respectively over the next six months. This probably indicated an urgent need for further intervention as it was felt that the impact of the Phase I intervention was waning.

## Phase II intervention programme

The Phase II intervention programme was initiated in July 2010 as the Antimicrobial Stewardship Programme based on IDSA (Infectious Diseases Society of America) guidelines [16]. This stage comprised the formation and effective functioning of the antimicrobial stewardship committee. The function of this committee was to optimize clinical outcome and minimize unintended consequences of antimicrobial use, namely toxicity and selection of drug-resistant pathogens, and to modify existing antibiotic guidelines as required depending on the antibiograms and discussion with physicians. It also included a prospective audit with intervention, feedback, formulary restriction and preauthorization which were implemented in combination with rigorous infection control policies and protocols to prevent the further spread of multi-resistant pathogens. Similar components of antimicrobial stewardship interventions were recommended by Patel et al. [17]

The impact of stewardship programs on antimicrobial use has been summarized in many study reviews [18,19]. In our study, a significant decrease of 24.7% was only observed in the case of carbapenem-resistant *Pseudomonas* in the nine-month period from July 2010 to March 2011. The rest of the resistance patterns remained fairly stable, possibly indicating that a more prolonged time period is required to have an impact. It may also indicate the natural evolution of antimicrobial resistance.

The results of Gram-positive organisms, namely MRSA, MRSE and VRE, did not show any significant correlation with the intervention programmes, and considering that they do not form a major part of the total isolates, their in-depth analysis is beyond the purpose of this paper. However, their detailed results are shown in Additional file 1: Table S1.

## Correlation of antimicrobial consumption with resistance

ICU is a high antibiotic usage area; therefore, a correlation was simultaneously sought between antibiotic usage [antibiotic prescription (DDD/1000 in-patient days)] and resistance patterns in the phase II post-intervention period (July 2010 to March 2011) (Table 4). A positive correlation was observed with carbapenem (imipenem and meropenem) and cefoperazone-sulbactam usage and the development of resistance in *Acinetobacter* and *Pseudomonas*. However, Piperacillin-tazobactam showed a positive correlation with *Acinetobacter* but a negative correlation with *Pseudomonas*.

**Table 4:** Correlation analysis between Antibiotic prescription and Antimicrobial resistance (July 2010-March 2011) in ICU

**Organism(s) and Drug resistance**	**r (Correlation Coefficient)**	**Interpretation of correlation**
***E.coli + Kleb***		
Imepenem	−0.29	Negative
Meropenem	−0.39	Negative
CSL	0.03	Positive
Pip/Tazo	0.01	Positive
***Acinetobacter***		
Imepenem	0.17	Positive
Meropenem	0.29	Positive
CSL	0.48	Positive
Pip/Tazo	0.33	Positive
***Pseudomonas***		
Imepenem	0.04	Positive
Meropenem	−0.6	Negative
CSL	0.62	Positive
Pip/Tazo	−0.39	Negative

**Figure 1:**
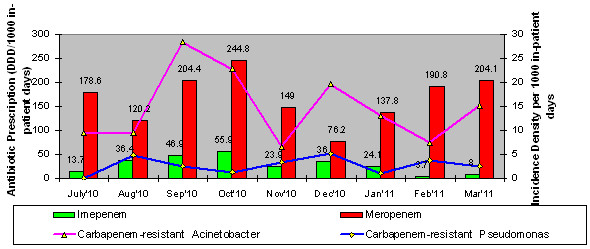
Antimicrobial Resistant A cinetobacter & Pseudonomas isolates and respective Carbapenem prescription volumes form Jul'10 to Mar'11 in ICU.

**Figure 2:**
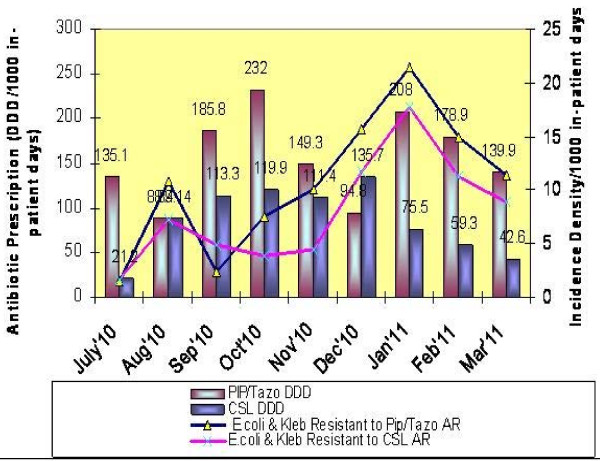
Antimicrobial Resistant E, coli & Kleb isolates (%) and respective Piperacillin/Tazobactam & Cefoparazone/Sulbactam Antibiotic prescription volume form Jul'10 to Mar'11 in ICU.

In *E.coli* and *Klebsiella,* a positive correlation was observed with the usage of cefoperazone-sulbactam and piperacillin-tazobactam, however a negative correlation was seen with the usage of carbapenems (imipenem and meropenem) and the development of resistance to these antibiotics (see Figures 1 and 2). There are several possible explanations for the lack of significant correlation between antibiotic prescription and resistance in our study. As may be pointed out, resistance selection pressure occurs at the individual level and calculating antibiotic prescription using DDD measurements does not measure individual exposure to antibiotics [20].

In conclusion, our data demonstrated correlation between antibiotic prescription and Gram-negative bacterial resistance to several, but not all, key antimicrobial agents in the hospital. In areas where Gram-negative bacterial resistance is endemic and prescription of broad-spectrum antimicrobial agents is high, factors other than antimicrobial usage may be equally important in maintaining high resistance rates [21].

The natural march of resistance and the entry of more drug-resistant organisms in the tertiary care centre also play a role in increasing overall resistance percentages.

Our study highlights the increasing resistance in Gram-negative bacteria towards antibiotics in our hospital. As this study was limited to the antibiotic-resistant bacteria isolated from patients in the tertiary care hospital, the true extent of resistance to these agents among bacterial isolates from community-acquired infections is not clear. Also an attempt should be made to risk-stratify the patients into three types; type 1 being patients who have had no prior antibiotic treatment or contact with the healthcare system, young patients with few co-morbid conditions, Type 2 as recent admission with short antibiotic therapy and older patients and Type 3 as long hospitalization, multiple antibiotic therapies and immunocompromised state.

Antimicrobial stewardship programs are clearly required in hospitals to promote and emphasize the rational use of antimicrobials. Although resistance is a worldwide concern, it is first and foremost a local problem: selection and amplification of resistant members of a species are occurring in individual hospitals (and communities), which can then spread worldwide [22]. An effective infection control program can make a significant contribution to limiting the spread of resistance. However, in the present study, the gaps in the infection control program due to the higher turnover rate of the nursing staff, regular admission of patients infected with resistant strains into the hospital and the high endemicity of drug-resistant pathogens in the region could explain the limited impact of the antimicrobial stewardship actions undertaken in this hospital.

## Competing interests

No potential conflict of interest in terms of financial and other relationships exists for any of the three authors (Namita Jaggi, Pushpa Sissodia and Lalit Sharma).

## Authors’ contributions

NJ conceived the study, and participated in its design and coordination and helped to draft the manuscript. PS participated in the design of the study, data collection and provided writing assistance and statistical analysis. LS provided technical assistance for the specimen processing, identification and susceptibility testing of bacterial isolates. All authors read and approved the final manuscript.

## Supplementary Material

Additional file 1 **Table S1. **Gram Positive Culture data of various samples from July 2007 to March 2011. Click here for file
